# Low-Power Resistive Switching Characteristic in HfO_2_/TiO_x_ Bi-Layer Resistive Random-Access Memory

**DOI:** 10.1186/s11671-019-2956-4

**Published:** 2019-05-09

**Authors:** Xiangxiang Ding, Yulin Feng, Peng Huang, Lifeng Liu, Jinfeng Kang

**Affiliations:** Institute of Microelectronics, Peking University, Beijing, 100871 China

**Keywords:** RRAM, Low power, Atomic layer deposition, Titanium oxide

## Abstract

Resistive random-access memory devices with atomic layer deposition HfO_2_ and radio frequency sputtering TiO_x_ as resistive switching layers were fabricated successfully. Low-power characteristic with 1.52 μW set power (1 μA@1.52 V) and 1.12 μW reset power (1 μA@1.12 V) was obtained in the HfO_2_/TiO_x_ resistive random-access memory (RRAM) devices by controlling the oxygen content of the TiO_x_ layer. Besides, the influence of oxygen content during the TiO_x_ sputtering process on the resistive switching properties would be discussed in detail. The investigations indicated that “soft breakdown” occurred easily during the electrical forming/set process in the HfO_2_/TiO_x_ RRAM devices with high oxygen content of the TiO_x_ layer, resulting in high resistive switching power. Low-power characteristic was obtained in HfO_2_/TiO_x_ RRAM devices with appropriately high oxygen vacancy density of TiO_x_ layer, suggesting that the appropriate oxygen vacancy density in the TiO_x_ layer could avoid “soft breakdown” through the whole dielectric layers during forming/set process, thus limiting the current flowing through the RRAM device and decreasing operating power consumption.

## Introduction

Resistive random-access memory (RRAM) provides a promising solution for scaling down beyond traditional charge-based memory due to simple cell structure, non-volatile storage, high-speed operation, and high on/off ratio [1–10]. Recently, One-transistor one-resistor (1T1R) is a widely accepted structure to prevent inaccurate resistance measurements caused by a sneak path current in 1R array [11, 12]. Besides, the emerging memory, especially oxide-based RRAM, has been proposed for plastic synaptic devices due to the gradual conductance modulation with pulse number, which can mimic biological synaptic behavior to receive signals from pre- and postsynaptic neuron [13–17]. However, high resistive switching current is the main limitation for low-power and high-density application [18–20]. The 1T1R array also faces scaling challenges if the operation current of RRAM cannot scale accordingly. For example, when the CMOS technology is scaling down to 27 nm, the drive current will decrease to 40 μA [21]. Therefore, reducing operation current of RRAM device down to 10 μA by optimizing structure and material is necessary to continue 1T1R scaling [22]. In addition, biological synapses consume around 1 ~ 10 fJ per event in the complex human brain, thus, reducing the energy consumption of electrical synaptic devices as little as biological synapses is important for the development of neuromorphic artificial neural networks (ANNs) [23–25]. Therefore, limiting the device current and reducing the power consumption will benefit the practical process for data storage and neuromorphic computing application.

In this work, Pt/HfO_2_/TiO_x_/Pt devices with a different oxygen content of TiO_x_ film were fabricated, and low-power characteristic in low oxygen content was demonstrated. The memory device achieved 1.52 μW set power and 1.12 μW reset power through decreasing oxygen content of the TiO_x_ film during the sputtering process. The conductive mechanism for low-power characteristic was analyzed further to provide insights into oxide RRAM design.

## Methods

**Fig. 1:**
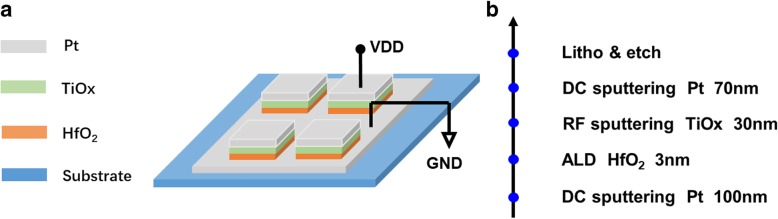
**a** The structure of Pt/HfO2/TiO_x_/Pt device. **b** The fabrication process flow

**Fig. 2:**
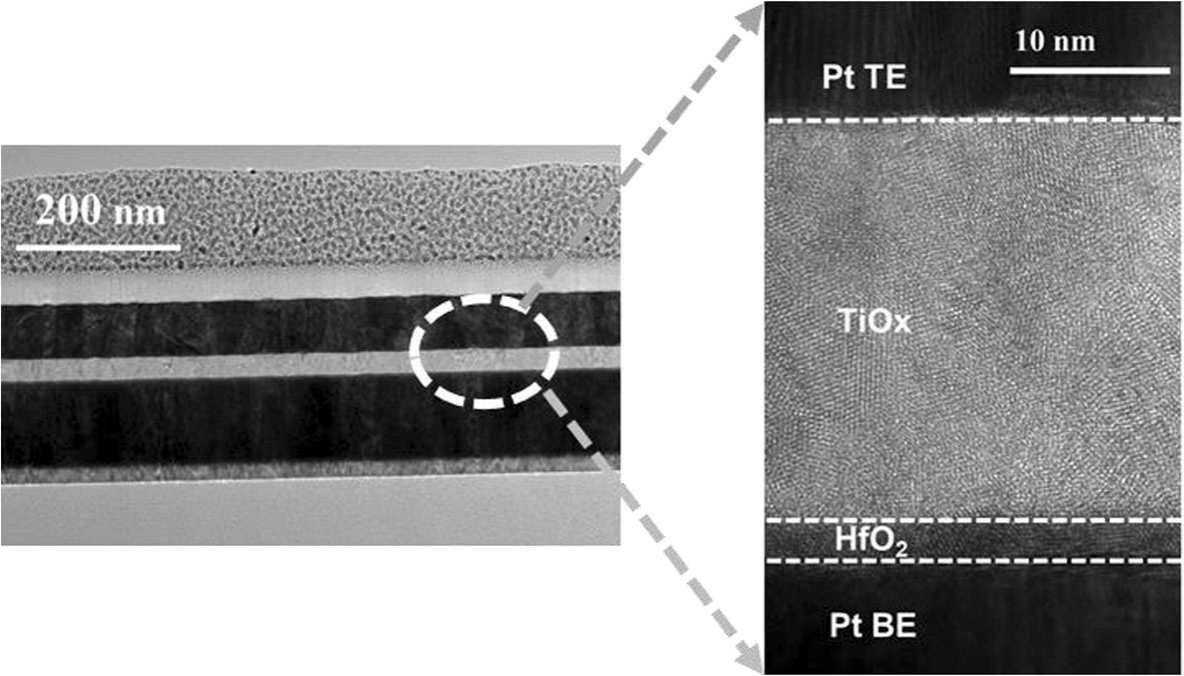
TEM cross-sections of the Pt/HfO_2_/TiO_x_/Pt device

The Pt/HfO_2_/TiO_x_/Pt device structure and fabrication process are shown in Fig. 1a and b. At first, on Si/SiO_2_/Ti substrate, a 100-nm Pt bottom electrode (BE) was prepared by direct current (DC) sputtering at room temperature. Next, 3 nm HfO_2_ was deposited by atomic layer deposition (ALD) (Picosun R200) technique at 300 °C using TEMAH and H_2_O as precursors. Subsequently, 30 nm TiO_x_ was deposited with different oxygen content by radio frequency sputtering. During TiO_x_ film sputtering process, fixing the total gas flow of argon (Ar) and oxygen (O_2_) as 20 sccm and changing the oxygen partial pressure with 9%, 11%, and 13%, three sample devices (S1, S2, and S3) were obtained to investigate the influence of oxygen content of TiO_x_ film on the resistive switching performance. Following that, a 70-nm Pt top electrode (TE) was deposited by DC sputtering and patterned with lithography. Finally, 100 μm × 100 μm square-shape devices were formed by reactive ion etching (RIE). Bias voltage was applied on the TE, and the BE was connected with the ground. The high-resolution transmission electron microscope (HRTEM) images of the cross-section of the Pt/HfO_2_/TiO_x_/Pt are shown in Fig. 2. The electrical characteristics of the devices were measured with Agilent B1500A semiconductor parameter analyzer. The chemical states of atoms in the TiO_x_ films were investigated by X-ray photoelectron spectroscopy (XPS, Axis Ultra).

## Results and Discussion

**Fig. 3:**
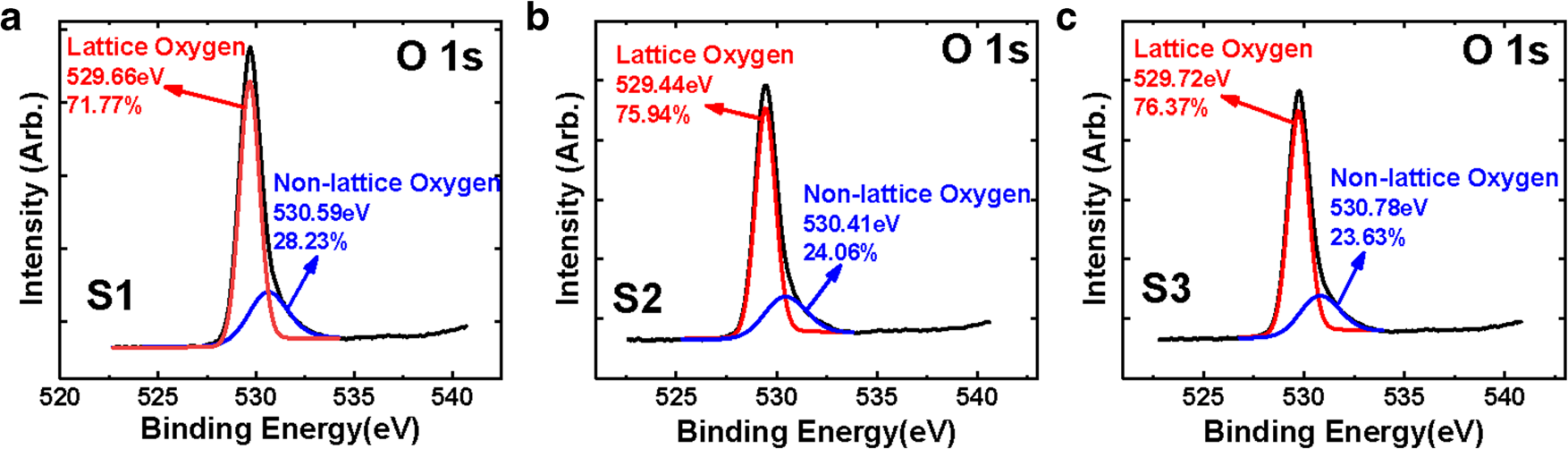
O 1s XPS scan spectra of TiO_x_ films in S1, S2, and S3. Oxygen partial pressure was set as **a** 9%, **b** 11%, and **c** 13% during TiO_x_ film sputtering process

Figure 3a, b, and c show the XPS O 1s core-level spectra of TiO_x_ films. To clarify the chemical bond of oxygen in the films, the asymmetric O 1s peaks are divided into two peaks, which are generally ascribed to the O^2−^ bonded by metal ions and O^2−^ in the oxygen-deficient region [26]. Oxygen partial pressure during TiO_x_ film sputtering process was set as 9%, 11%, and 13%, respectively, and the corresponding oxygen-deficient content in three samples is about 28.23%, 24.06%, and 23.63%, indicating that the content of non-lattice oxygen ions and oxygen vacancies decreases with increasing oxygen partial pressure.

**Fig. 4:**
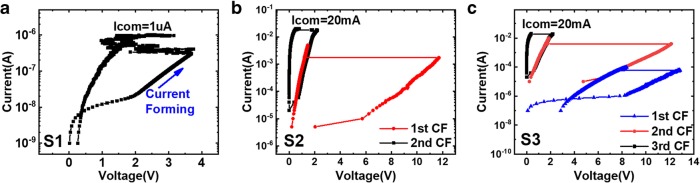
Current forming process of the Pt/HfO2/TiO_x_/Pt RRAM device in **a** S1, **b** S2, and **c** S3

For the fresh devices, the original state is in high resistance state (HRS). As shown in Fig. 4, current forming (CF) is applied to initiate the formation of the conductive filament and change the device state to low resistance state (LRS) [27]. When applying 1 μA of current compliance, a conductive path is formed in S1 and the stable set/reset process can be achieved in the subsequent operation. For S2 and S3, reset operation is not successful from the middle state of the device during CF process until the current compliance is up to 20 mA.

**Fig. 5:**
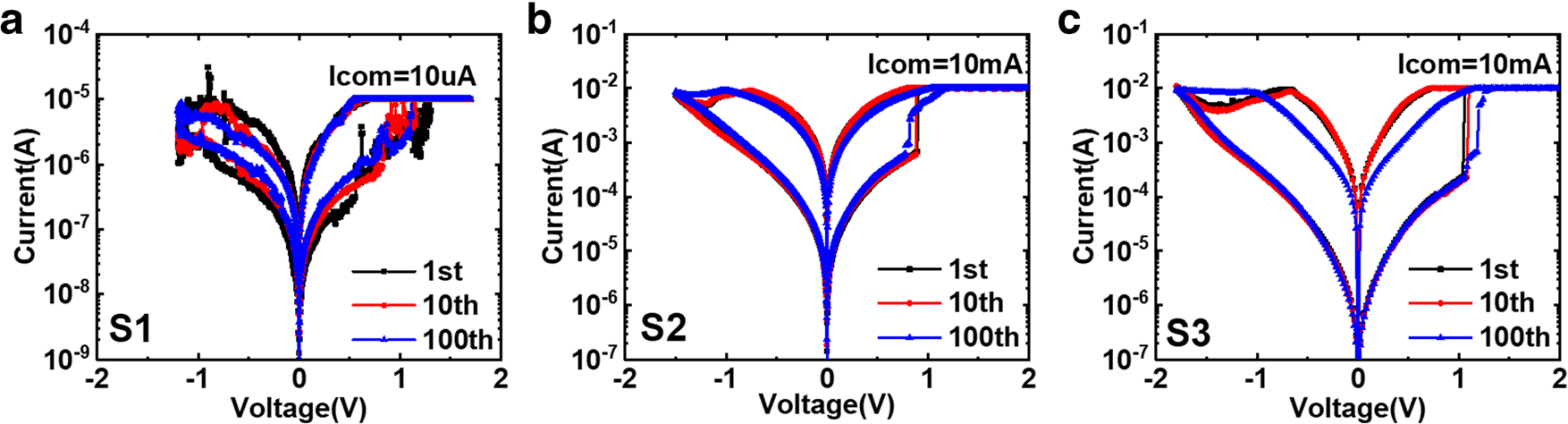
100 cycles stable bidirectional I-V curves in **a** S1, **b** S2, and **c** S3

In order to test the electrical performance of the RRAM device, DC measurements under voltage sweep are carried out. Positive bias voltage in forming and set process is applied on TE to complete the conductive filament, and negative bias voltage in reset process is to break the filament. When the bias is swept back and forth, 100 cycles of bipolar switching current-voltage (I-V) curves of three samples are shown in Fig. 5. The S1 devices achieve stable resistive switching performance under 10 μA current compliance, but the operation current is up to 10 mA for the other two samples. The low-power characteristic of S1 could be attributed to high oxygen vacancy content preexisting in TiO_x_ film, which limits the current effectively during forming/set process.

**Fig. 6:**
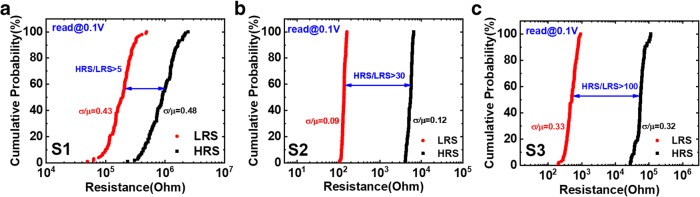
Cycle-to-cycle variation of R_LRS_ and R_HRS_ for 100 cycles in **a** S1, **b** S2, and **c** S3

**Fig. 7:**
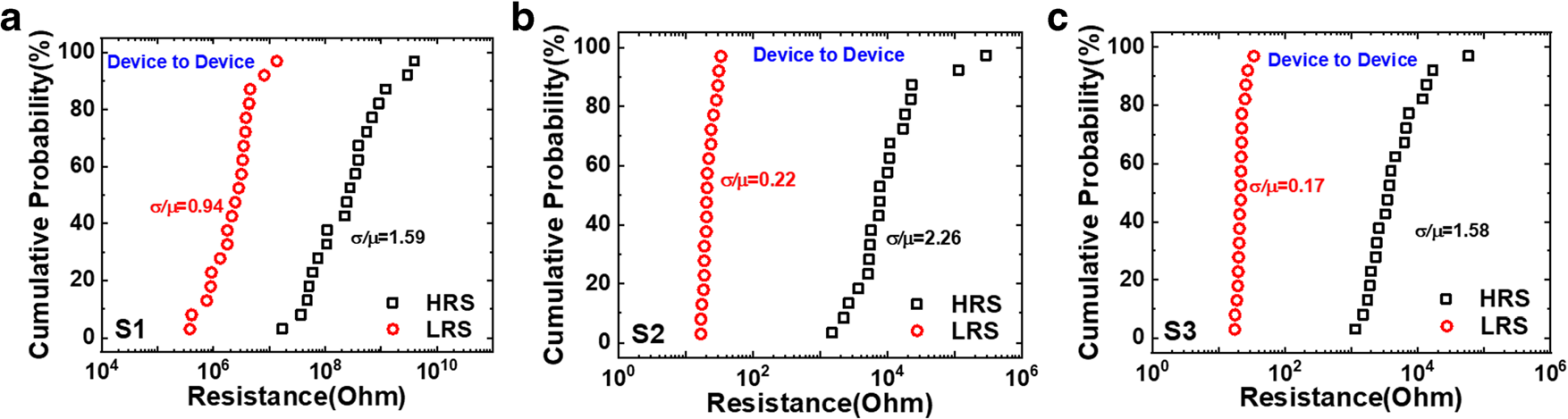
Device-to-device variation of R_LRS_ and R_HRS_ for 20 devices in **a** S1, **b** S2, and **c** S3

Figures 6 and 7 depict the cycle-to-cycle and device-to-device variation (relative standard deviation, (*σ*/*μ*)) of three samples, and the statistics are summarized in Tables 1 and 2. For S1, weak hopping current causes modest resistance distribution, and the strong conductive filaments formed in S2 and S3 guarantee the relatively stable resistance distribution. Although there is a little degradation for S3 after dozens of cycles, the on/off ratio is still over 100.

**Table 1:** The cycle-to-cycle variation characteristic of three samples

Sample	Average value(*μ*) (ohm)		Standard deviation(*σ*) (ohm)		Relative standard deviation (*σ*/*μ*)	
	LRS	HRS	LRS	HRS	LRS	HRS
S1	1.96e5	9.91e5	8.35e4	4.80e5	0.43	0.48
S2	134.75	5.35e3	12.13	663.24	0.09	0.12
S3	520.12	5.83e4	170.40	1.85e4	0.33	0.32

**Table 2:** The device-to-device variation characteristic of three samples

Sample	Average value(*μ*) (ohm)		Standard deviation(*σ*) (ohm)		Relative standard deviation(*σ*/*μ*)	
	LRS	HRS	LRS	HRS	LRS	HRS
S1	3.20e6	6.28e8	2.99e6	9.96e8	0.94	1.59
S2	22.32	2.90e4	4.83	6.56e4	0.22	2.26
S3	22.09	7.61e3	3.67	1.20e4	0.17	1.58

**Fig. 8:**
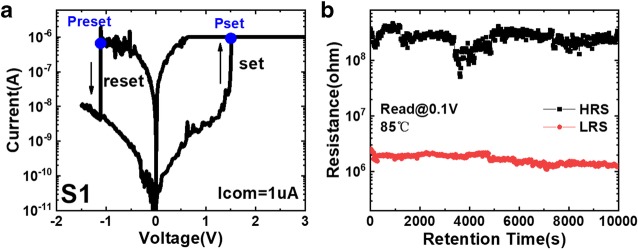
**a** Resistive switching performance under 1 μA current limitation. **b** Retention characteristic in S1 is over 10^4^ s under 85 °C

As shown in Fig. 8, the set power (Pset) 1.52 μW and the reset power (Preset) 1.12 μW are reached under a low compliance current of 1 μA. The power consumption of the other two samples is tens of milliwatt due to 10 mA of operation current. Besides, the resistance states of S1 can keep retention characteristics over 10^4^ s under 85 °C with approximate 100 on/off ratio, which is stable for data storage application.

**Fig. 9:**
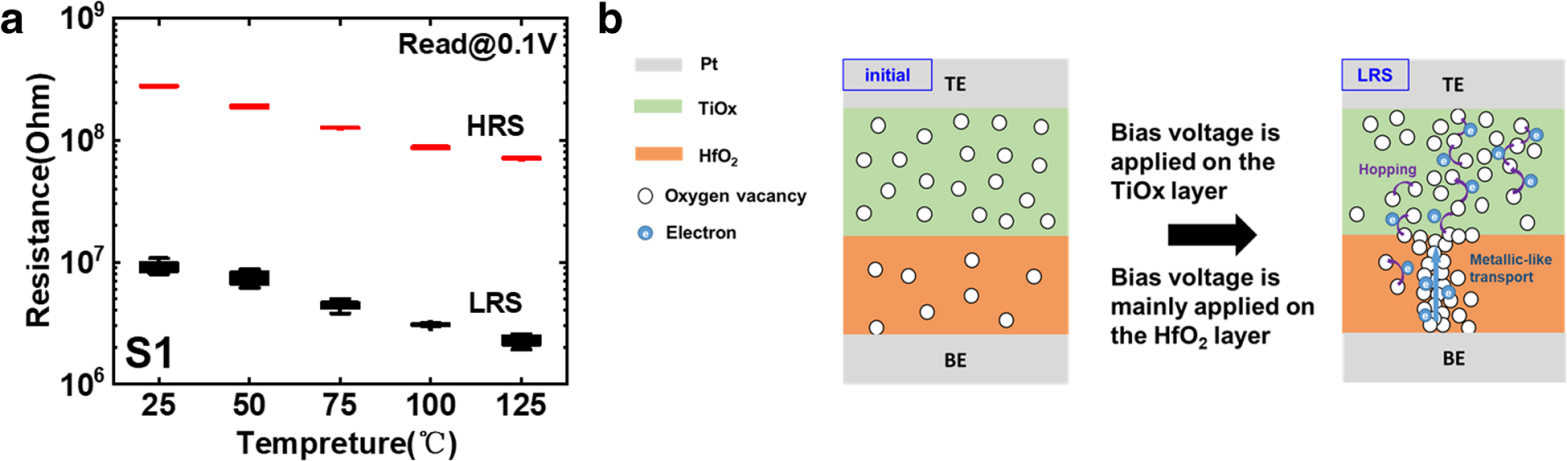
**a** The resistance changes with temperature in S1. **b** The corresponding schematic diagram of conductive mechanism

**Fig. 10:**
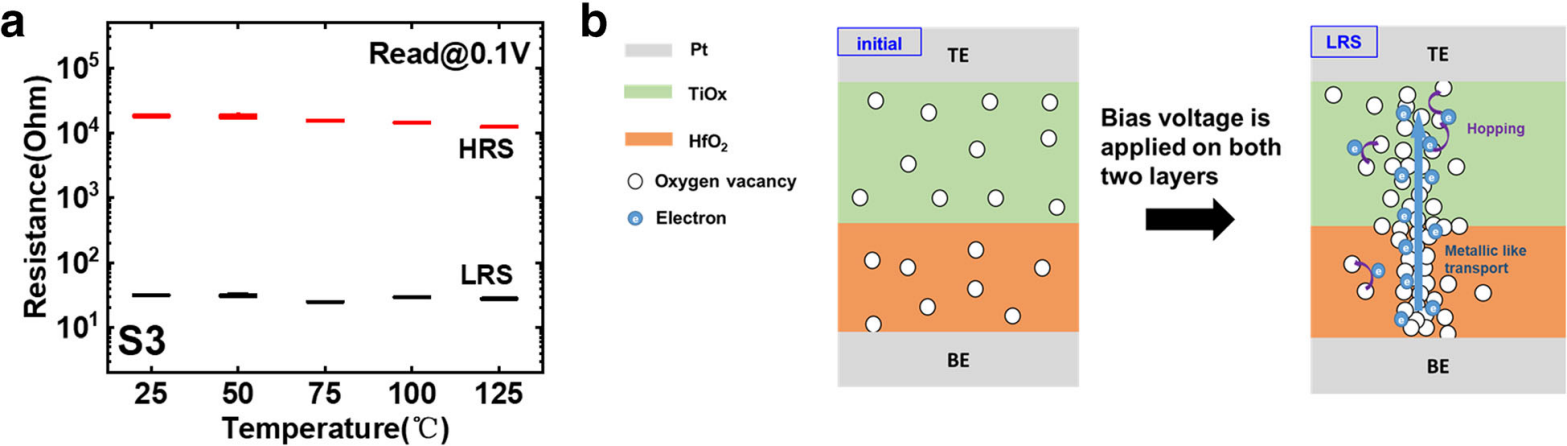
**a** The resistance changes with temperature in S3. **b** The corresponding schematic diagram of conductive mechanism

**Fig. 11:**
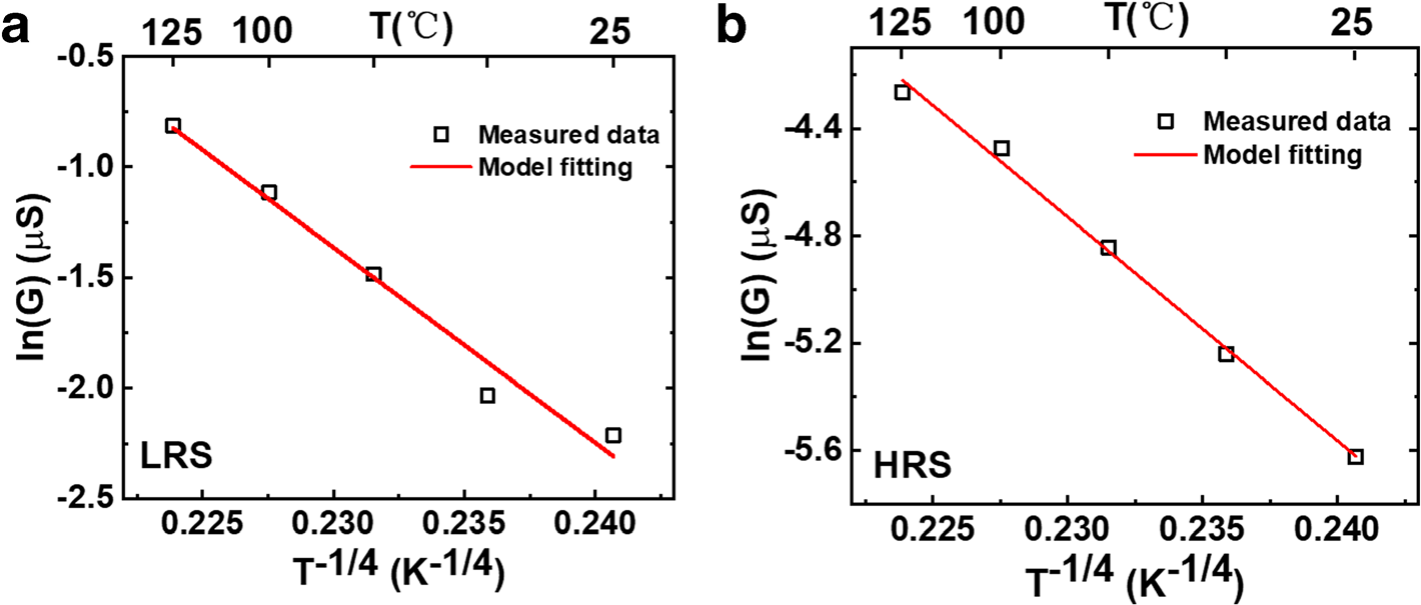
Temperature dependence of the conductance of S1 in **a** LRS and **b** HRS

To elucidate the conductive mechanism of low-power characteristic, we carried out temperature measurements for S1 and S3 with different operation current and investigated the corresponding mechanism, as shown in Figs. 9 and 10. From 25 °C to 125 °C, the resistance of S1 decreases with temperature, but the resistance of S3 almost does not change. The experimental conductance and temperature are fitted with Mott’s variable range hopping model [28], as shown as Fig. 11, which indicates that the main conductive mechanism of S1 is electrons hopping via localized oxygen vacancy defects in dielectric oxide [29]. When decreasing the oxygen partial pressure during the TiO_x_ sputtering process, as shown in S1, the oxygen vacancy content in initial TiO_x_ layer increases and the film resistance decreases [30]. The voltage on the TE is applied mainly on the HfO_2_ layer and a mass of oxygen vacancies are motivated to form the conductive filament. After that, new oxygen vacancies are also motivated in the TiO_x_ layer, but the connection among oxygen vacancies is weak. Therefore, electrons hopping conduction in TiO_x_ is dominant, which ensures low 1-μA resistive switching current.

However, as shown in Fig. 10, after increasing oxygen content during TiO_x_ sputtering process, HRS and LRS remain almost unchanged with temperature, which is most likely associated with the metallic-like transport mechanism, induced by electrons transport through conductive filament consisted of concentrated oxygen vacancies. Compared with the RRAM device of S1, fewer oxygen vacancies in initial TiO_x_ layer of S3 are not enough for electrons hopping conduction. Besides, due to the increased resistance of TiO_x_ film, the voltage bias is applied on both HfO_2_ layer and TiO_x_ layer at the same time. Electric initialization leads to plenty of oxygen vacancies motivated in HfO_2_ and TiO_x_ layers. These oxygen vacancies form a strong conductive filament in both two dielectric layers, and the abundant extended electrons flow through the two adjacent oxygen vacancies, which causes high operation current during resistive switching process.

In principle, it is possible to control oxygen content carefully to achieve low-power performance in other oxide resistive memories (OxRRAM) related to the oxygen vacancy. The requirement for the oxide layer is that there should be enough oxygen vacancies in the initial state for electrical hopping conduction in case of the device breakdown. However, the excessive oxygen vacancies will cause unstable endurance characteristic and deteriorate the device performance. Therefore, the appropriate oxygen vacancies are necessary to limit operation current and decrease power consumption.

Table 3 compares some of the key parameters of the Pt/HfO_2_/TiO_x_/Pt device with other recent reports. The device has important merits of low 1.12 μW resistive switching power and over 100 HRS/LRS ratio among various RRAM devices.

**Table 3:** Comparison of device performance for RRAM devices

Device structure	Type	Iset@Vset	Ireset@Vreset	HRS/LRS	Pset, preset	Reference
Pt/TiO_x_/HfO_2_/Pt	OxRRAM	1 μA@1.52 V	1 μA@− 1.12 V	100	1.52 μW, 1.12 μW	This work
Pt/C/Ta_2_O_5_/TiN	OxRRAM	1 mA@− 1.5 V	0.3 mA@2.6 V	100	1.5 mW, 0.78 mW	[5]
TiN/Ti/HfO_x_/TiN	VRRAM	100 μA@1.15 V	92 μA@− 0.98 V	10	115 μW, 90.2 μW	[6]
Cu/black phosphorus/Au	CBRAM	0.9 mA@0.71 V	0.9 mA@− 0.57 V	1000	0.64 mW, 0.51 mW	[7]
Sn/HfO_2_/Pt	CBRAM	1 mA@3.5 V	6 mA@− 1.67 V	1e5	3.5 mW, 10.02 mW	[8]
Nb/NiO/Nb	Unipolar	15 mA@0.82 V	15 mA@0.38 V	100	12.3 mW, 5.7 mW	[9]
Ta/Ta_2_O_5_/Pt	Unipolar	1 mA@2.31 V	8 mA@1 V	20	2.31 mW, 8 mW	[10]

## Conclusions

In this work, 1-μA resistive switching current was demonstrated in Pt/HfO_2_/TiO_x_/Pt structure device. For the conductive mechanism, electrons hopping conduction is dominant in low oxygen content of the TiO_x_ layer, which limits operation current and decreases power consumption. Metallic-like transport is dominant in high oxygen content of the TiO_x_ layer, and “soft-breakdown” of two dielectric layers causes high operation current and high power consumption. The appropriate oxygen content of TiO_x_ film can limit the RRAM current and contribute to low-power characteristic, which provides a solution for large operation current and high-power issues and shows the promise for future embedded non-volatile memories and the Internet of things (IoT) applications.

## Abbreviations

1T1R: One-transistor one-resistor
ALD: Atomic layer deposition
ANNs: Artificial neural networks
BE: Bottom electrode
CF: Current forming
HRS: High resistance state
HRTEM: High-resolution transmission electron microscope
IoT: Internet of things
I-V: Current-voltage
LRS: Low resistance state
On/off ratio: HRS/LRS
OxRRAM: Oxide resistive memory
Preset: Reset power
Pset: Set power
RIE: Reactive ion etching
RRAM: Resistive random-access memory
TE: Top electrode
XPS: X-ray photoelectron spectroscopy
*σ*/*μ*: Relative standard deviation
